# Association between dietary fatty acid intake and preserved ratio impaired spirometry in U.S. adults: a population-based cross-sectional study

**DOI:** 10.3389/fnut.2025.1622795

**Published:** 2025-07-30

**Authors:** Xiang Liu, Wei Zeng, Wangyan Zhou, Dayang Zheng, Xu Yang, Weijun Liao

**Affiliations:** ^1^Department of Thoracic Surgery, The Second Affiliated Hospital, Hengyang Medical School, University of South China, Hengyang, China; ^2^Department of Medical Record, The First Affiliated Hospital, Hengyang Medical School, University of South China, Hengyang, China

**Keywords:** dietary fatty acid, PRISm, NHANES, lung function, nutrition

## Abstract

**Background:**

Preserved ratio impaired spirometry (PRISm) is increasingly recognized as a clinically relevant but underdiagnosed lung function abnormality. This pulmonary phenotype is clinically significant yet remains insufficiently studied. Although dietary fatty acids are known to have anti-inflammatory and immune-regulating properties, their relationship with PRISm has not been previously explored. This study aimed to evaluate the associations between intake of saturated (SFA), monounsaturated (MUFA), and polyunsaturated fatty acids (PUFA) and the prevalence of PRISm in U.S. adults.

**Methods:**

We conducted a cross-sectional analysis using data from 9,103 adults in the 2007–2012 National Health and Nutrition Examination Survey (NHANES). Dietary intake of SFA, MUFA, and PUFA was assessed from two 24-h dietary recalls. Fatty acid variables were log-transformed and standardized. Logistic regression models were used to estimate odds ratios (ORs) and 95% confidence intervals (CIs) for the associations between fatty acid intake and PRISm, adjusting for sociodemographic, behavioral, and clinical covariates. Nonlinear relationships were examined using restricted cubic splines. A two-sided *p*-value <0.05 was considered statistically significant.

**Results:**

Among the study population (mean age 45.6 ± 15.8 years; 47.7% male), 1,362 participants (15.0%) exhibited the PRISm phenotype. In models controlling for demographic, lifestyle, and clinical variables, each standard deviation increase in SFA [0.86 (0.75–0.99)] and PUFA [0.88 (0.79–0.99)] intake was associated with a statistically significant reduction in the odds of PRISm. MUFA intake was not significantly related to PRISm. Restricted cubic spline analysis indicated no evidence of non-linearity in these associations. The inverse relationships for SFA and PUFA were also consistent across demographic and clinical subgroups.

**Conclusion:**

Greater consumption of saturated and polyunsaturated fatty acids was associated with a lower prevalence of PRISm in a nationally representative adult population. These associations were consistent across key demographic and clinical subgroups. If confirmed in prospective studies, our findings may inform early dietary strategies to support pulmonary health.

## Graphical abstract

Created with BioRender.com.

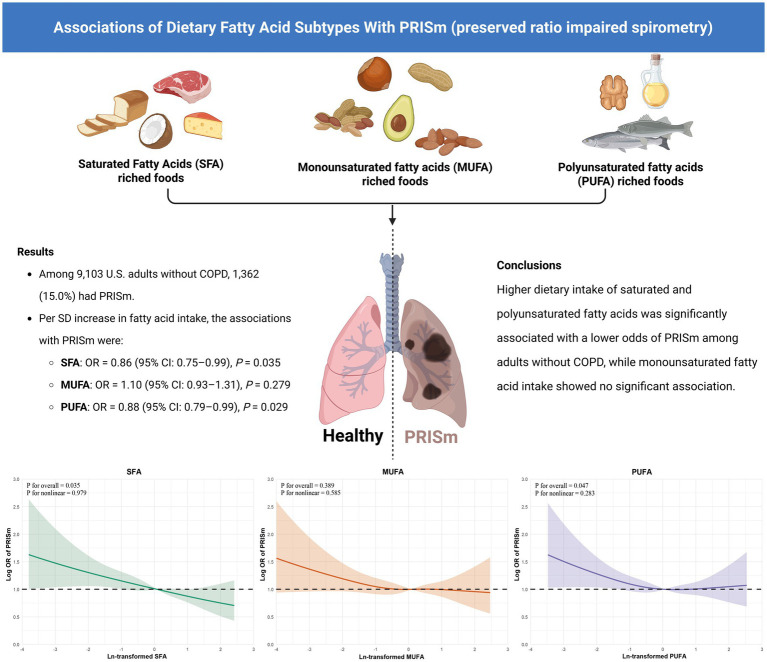

## 1 Background

Preserved ratio impaired spirometry (PRISm) is identified when expiratory function is reduced, yet the ratio of forced expiratory volume in one second (FEV₁) to forced vital capacity (FVC) remains within normal limits, indicating a non-obstructive yet clinically relevant pulmonary pattern, as outlined by the American Thoracic Society guidelines (1). Emerging evidence has shown that PRISm is clinically significant, associated with increased mortality risk—especially from cardiovascular and respiratory causes—as well as impaired functional status, systemic inflammation, and heightened likelihood of progression to chronic obstructive pulmonary disease (COPD) (2–5). However, despite these serious prognostic implications, PRISm remains an underrecognized clinical entity, and its underlying risk factors and pathophysiological mechanisms remain poorly defined. Identifying modifiable risk factors for PRISm represents a critical unmet clinical need.

Diet is a potentially modifiable determinant of pulmonary health, and dietary fatty acids—particularly saturated (SFA), monounsaturated (MUFA), and polyunsaturated fatty acids (PUFA)—have drawn attention for their immunomodulatory and pro- or anti-inflammatory properties (6–8). Prior studies have primarily explored associations between fatty acid intake and respiratory outcomes such as asthma and COPD, often focusing narrowly on specific fatty acid subclasses (particularly omega-3 polyunsaturated fatty acids) or individual food groups (e.g., fish oil, nuts, or processed meats) (9–13). While these studies indicate beneficial effects of certain dietary fats on respiratory health, they are limited by relatively small sample sizes, specific patient populations, and inadequate representation of the general population. Importantly, PRISm has not yet been systematically studied in relation to dietary factors, representing a major research gap.

Given the growing clinical recognition of PRISm and its significant public health burden (14, 15), we conducted this study using data from the National Health and Nutrition Examination Survey (NHANES) to examine whether dietary intake of saturated, monounsaturated, and polyunsaturated fatty acids is associated with PRISm in a nationally representative sample of U.S. adults. Based on prior evidence suggesting anti-inflammatory effects of specific fatty acids, we hypothesized that higher intake of saturated and polyunsaturated fatty acids would be independently associated with lower prevalence of PRISm. The specific objectives were to: (1) assess these associations while adjusting for demographic, lifestyle, and clinical factors; (2) examine potential nonlinear dose–response relationships and subgroup differences; and (3) explore biological plausibility through sensitivity analyses involving serum lipid biomarkers.

Clarifying how overall dietary fatty acid intake relates to early-stage non-obstructive pulmonary impairment (such as PRISm) could have important clinical and public health implications. Dietary fatty acids represent practical, modifiable targets for preventive strategies. Findings from this study may help inform dietary guidelines for maintaining respiratory health and support policy decisions aimed at reducing the burden of lung function impairment.

## 2 Methods

### 2.1 Study design and population

**Figure 1:**
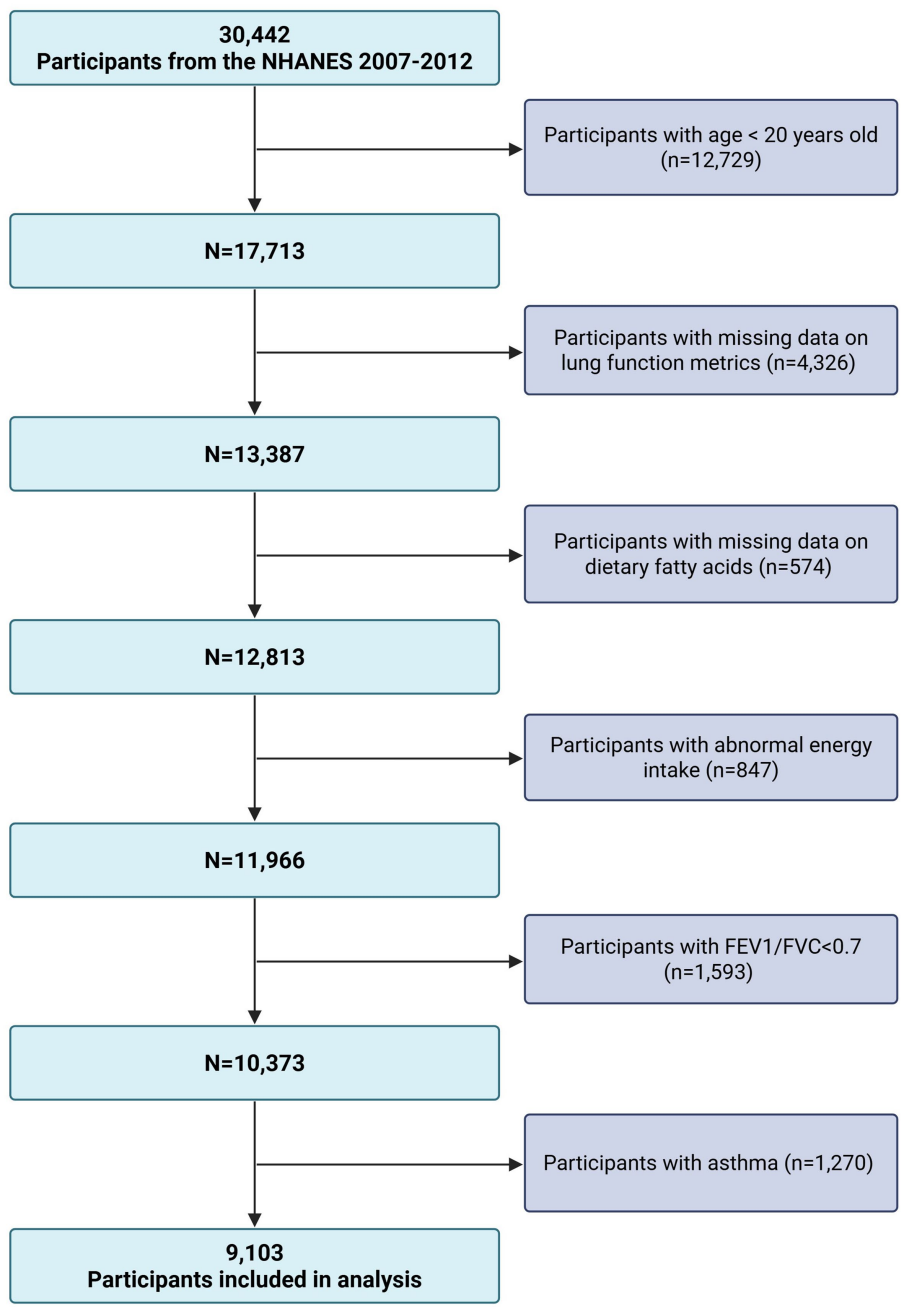
Flow chart of participant selection. NHANES, National Health and Nutrition Examination Survey; FEV, forced expiratory volume in one second; FVC, forced vital capacity.

This analysis was based on cross-sectional data from the 2007–2012 cycles of the (NHANES) a nationwide surveillance program conducted by the U.S. Centers for Disease Control and Prevention. Designed to reflect the health and nutritional characteristics of the U.S. civilian, non-institutionalized population, NHANES employs a complex, multistage probability sampling design that combines standardized interviews with comprehensive physical assessments (16). Of the 30,442 adult participants aged 20 years and older, exclusions were made for individuals lacking data on spirometry (*n* = 4,326) or dietary fatty acid intake (*n* = 574), reporting implausible daily energy intake (*n* = 847), or with incomplete covariate information. Participants with FEV1/FVC ratios below 0.70 (*n* = 1,593) or a history of asthma based on self-report (*n* = 1,270) were also removed. The final analytic cohort included 9,103 eligible adults (Figure 1).

### 2.2 Dietary fatty acid assessment

Dietary intake data were obtained from two non-consecutive 24-h dietary recall interviews administered to each participant. These recalls were conducted in person by trained interviewers using the Automated Multiple-Pass Method (AMPM), a validated five-step dietary assessment protocol developed by the U.S. Department of Agriculture (USDA) to improve recall accuracy and reduce bias. Each food and beverage item reported was linked to its corresponding nutrient composition using the USDA’s Food and Nutrient Database for Dietary Studies (FNDDS), which provides detailed fatty acid profiles per food item (17). For each participant, intakes of SFA, MUFA, and PUFA were estimated as the mean of the two 24-h recalls and expressed in grams per day (g/day). To account for confounding by total energy intake, we applied the residual method, a standard approach in nutritional epidemiology. Specifically, we performed linear regression of each fatty acid subtype on total energy intake and used the residuals from this model to represent energy-adjusted fatty acid intakes. These standardized values were used in all subsequent regression analyses.

### 2.3 Definition of PRISm

Participants were classified as having PRISm if their post-bronchodilator spirometry demonstrated an FEV₁/FVC ratio ≥0.70 in combination with an FEV₁ < 80% of predicted, based on American Thoracic Society recommendations (18, 19). The Global Lung Function Initiative equations were used to derive individual-specific FEV₁ predictions, accounting for age, sex, height, and race/ethnicity.

### 2.4 Covariates selection

Confounders were identified *a priori* based on previous literature and biological plausibility (20, 21), included age, sex, race/ethnicity, education level, poverty-to-income ratio (PIR), body mass index (BMI), smoking status, alcohol consumption, hypertension, diabetes, and cancer history. All covariates were derived from standardized NHANES questionnaires or physical assessments. The NHANES questionnaires are developed and maintained by the U.S. National Center for Health Statistics and are publicly accessible at https://wwwn.cdc.gov/nchs/nhanes/default.aspx. These instruments are rigorously tested for reliability and validity through pilot studies, cognitive testing, and expert review prior to implementation. No modifications were made to the questionnaires during the data collection process. Detailed definitions and classifications of all covariates are provided in Supplementary Table S1.

### 2.5 Statistical analysis

All analyses accounted for the complex, multistage sampling design of NHANES by incorporating survey weights, stratification, and clustering variables, thereby producing estimates representative of the U.S. population. Continuous variables were described using weighted means and SD, while categorical variables were presented as frequencies with corresponding weighted percentages. Between-group comparisons (PRISm vs. non-PRISm) were conducted using *t*-tests or Rao-Scott chi-square tests, as appropriate. Relationship between dietary fatty acid intake with PRISm were evaluated using logistic regression models. Fatty acid intakes were natural-log transformed and standardized to z-scores (per SD increase) to improve model performance and comparability. Three progressively adjusted logistic regression models were developed. Model 1 included no covariates. Model 2 accounted for basic demographic factors, including age, sex, and race. Model 3 further incorporated socioeconomic status (education and PIR), BMI, lifestyle behaviors (smoking and alcohol consumption), total energy intake, and self-reported diagnoses of diseases. To assess potential nonlinear exposure–response relationships, restricted cubic spline functions with four knots (5th, 25th, 75th, 95th percentiles) were incorporated (22). Subgroup analyses were conducted across strata defined by demographic characteristics (age, sex, race, education), PIR, obesity, and the presence or absence of chronic diseases. In sensitivity analyses, we further examined whether overall nutritional status might confound the associations between specific fatty acid intake and PRISm. To this end, we evaluated the associations of total dietary fat intake and total energy intake with PRISm prevalence using logistic regression models, both unadjusted and adjusted for potential confounders. Additionally, to explore potential mechanistic pathways, we assessed the associations between each major fatty acid subtype (saturated fat, monounsaturated fat, and polyunsaturated fat) and key serum lipid biomarkers, including high-density lipoprotein cholesterol (HDL-C), low-density lipoprotein cholesterol (LDL-C), total cholesterol, triglycerides, and apolipoprotein B (ApoB). These models were adjusted for demographic, socioeconomic, lifestyle, and clinical covariates, as well as total energy intake.

Missing data (<17.2% for any covariate) were imputed using the *missForest* R package (version 1.5), which applies a non-parametric random forest–based imputation algorithm suitable for mixed-type data (23, 24). Specifically, the following covariates had missing values and were included in the imputation process: education level, PIR, BMI, hypertension, diabetes, cancer, smoking status, and alcohol use. The proportion of missingness ranged from 0.8 to 15.2%, with PIR exhibiting the highest missing rate due to non-response on household income. All statistical procedures were performed using R software (version 4.4.1; R Foundation for Statistical Computing, Vienna, Austria). A two-tailed *p*-value less than 0.05 was considered indicative of statistical significance.

## 3 Results

### 3.1 Participant characteristics

The final analytic sample consisted of 9,103 adults, with an average age of 45.6 years (SD: 15.8) (Table 1). Among them, 47.7% (*n* = 4,345) were men and 52.3% (*n* = 4,758) were women. A total of 1,362 individuals, accounting for 15.0% of the population, were identified as having PRISm based on spirometric criteria. Compared with individuals with normal spirometry, those with PRISm were older (49.4 ± 15.0 vs. 44.9 ± 15.9 years), more frequently identified as non-Hispanic Black (62% vs. 17%). PRISm participants also had lower educational attainment and household income, as reflected by education level and PIR (both *p* < 0.01).

**Table 1:** Baseline characteristics of the study population by PRISm status.

Characteristics	PRISm status			*p*-value
	Overall (*N* = 9,103)	Normal spirometry (*N* = 7,741)	PRISm (*N* = 1,362)	
Age, years	45.6 ± 15.8	44.9 ± 15.9	49.4 ± 15.0	<0.001
Sex				0.225
Male	4,345 (48%)	3,716 (48%)	629 (46%)	
Female	4,758 (52%)	4,025 (52%)	733 (54%)	
Race				<0.001
Other race	1,659 (20%)	1,572 (22%)	87 (7.3%)	
Mexican American	1,024 (12%)	938 (13%)	86 (7.2%)	
Non-Hispanic White	3,661 (44%)	3,387 (48%)	274 (23%)	
Non-Hispanic Black	1,944 (23%)	1,202 (17%)	742 (62%)	
Education				0.005
<High school	2,265 (25%)	1,923 (25%)	342 (25%)	
High school	2,024 (22%)	1,678 (22%)	346 (25%)	
>High school	4,814 (53%)	4,140 (53%)	674 (49%)	
PIR	2.6 ± 1.6	2.6 ± 1.6	2.4 ± 1.5	<0.001
BMI, kg/m^2^	29.1 ± 6.7	28.7 ± 6.4	31.1 ± 7.9	<0.001
Hypertension				<0.001
No	6,469 (71%)	5,690 (74%)	779 (57%)	
Yes	2,634 (29%)	2,051 (26%)	583 (43%)	
Diabetes				<0.001
No	8,238 (90%)	7,121 (92%)	1,117 (82%)	
Yes	865 (9.5%)	620 (8.0%)	245 (18%)	
Cancer				0.683
No	8,529 (94%)	7,249 (94%)	1,280 (94%)	
Yes	574 (6.3%)	492 (6.4%)	82 (6.0%)	
Smoking				0.011
No	5,414 (59%)	4,647 (60%)	767 (56%)	
Yes	3,689 (41%)	3,094 (40%)	595 (44%)	
Drinking				<0.001
No	2,807 (31%)	2,285 (30%)	522 (38%)	
Yes	6,296 (69%)	5,456 (70%)	840 (62%)	
Energy, kcal/day	2,049.2 ± 763.3	2,063.2 ± 763.2	1,969.4 ± 759.3	<0.001
Protein, g/day	79.7 ± 35.7	80.4 ± 35.9	75.8 ± 34.4	<0.001
Fat, g/day	252.5 ± 102.9	253.9 ± 102.9	244.5 ± 102.7	0.002
Carbohydrate, g/day	16.5 ± 9.6	16.8 ± 9.7	14.9 ± 9.1	0.002
SFA, g/day	24.3 ± 13.6	24.6 ± 13.6	22.8 ± 13.4	<0.001
MUFA, g/day	27.5 ± 14.7	27.7 ± 14.7	26.6 ± 15.0	0.015
PUFA, g/day	17.2 ± 10.1	17.2 ± 10.1	16.8 ± 10.3	0.204

PRISm, preserved ratio impaired spirometry; PIR, poverty income ratio; BMI, body mass index; SFA, saturated fatty acids; MUFA, monounsaturated fatty acids; PUFA, polyunsaturated fatty acids. Continuous variables are presented as mean ± standard deviation. Categorical variables are presented as number (percentage). *p*-values were calculated using independent-sample *t* tests for continuous variables and chi-square tests for categorical variables.

In terms of health-related characteristics, individuals with PRISm had significantly higher BMI, as well as greater prevalence of chronic diseases, relative to those with normal lung function (both *p* < 0.001). Regarding dietary factors, PRISm participants consumed less total energy, protein, fat, and carbohydrates. Furthermore, intakes of SFA and MUFA were significantly lower in the PRISm group, while PUFA intake did not differ significantly.

**Figure 2:**
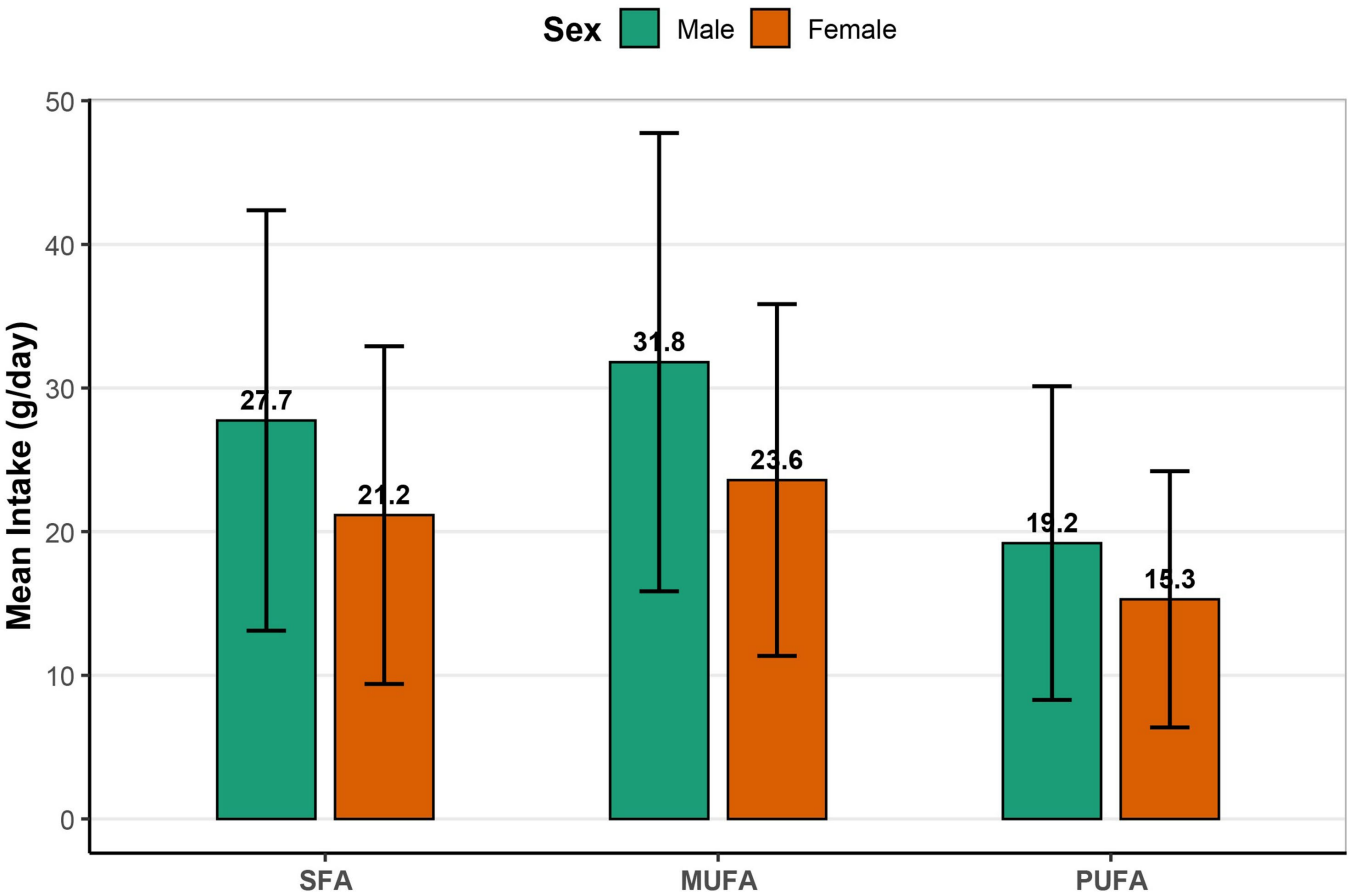
Mean daily intake of SFA, MUFA and PUFA by sex. Bar plots show mean intake (g/day) ± SD of SFA, MUFA and PUFA, stratified by sex (green = male; orange = female). SFA, saturated fatty acids; MUFA, monounsaturated fatty acids; PUFA, polyunsaturated fatty acids; SD, standard deviation.

As shown in Figure 2, men had consistently higher mean intakes of SFA (27.7 vs. 21.2 g/day), MUFA (31.8 vs. 23.6 g/day), and PUFA (19.2 vs. 15.3 g/day) compared to women.

### 3.2 Associations of fatty acid intake with PRISm

**Figure 3:**
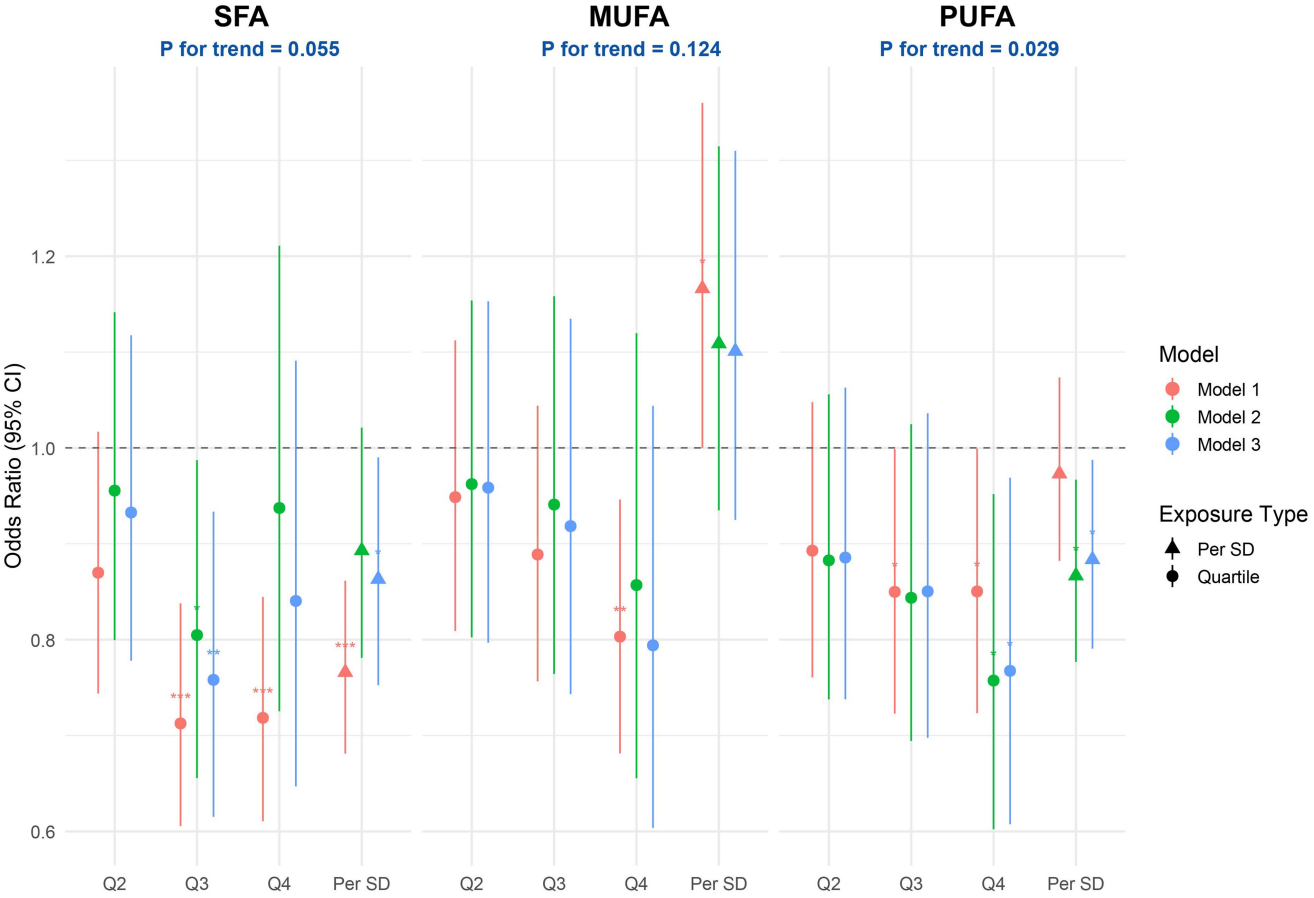
Associations between dietary fatty acids and PRISm. Forest plots display adjusted ORs and 95% CIs for PRISm according to quartiles (Q2–Q4 vs. Q1 reference) and per-SD increment of ln-transformed SFA, MUFA and PUFA. Model 1: none; Model 2: age, sex, and race; Model 3: Model 2 + energy intake, protein intake, carbohydrate intake, fat intake, education, BMI, PIR, smoking, alcohol drinking, hypertension, diabetes, and cancer. OR, odds ratio; CI, confidence interval; SD, standard deviation; PIR, poverty income ratio; BMI, body mass index.

Figure 3 illustrates the multivariable-adjusted associations between dietary fatty acid intake and the likelihood of PRISm. In the Model 3, greater consumption of SFA was linked to a statistically significant reduction in odds of PRISm prevalence, with higher intake of SFA, per standard deviation, was significantly linked to lower odds of PRISm (OR: 0.86; 95% CI: 0.75–0.99). Quartile-based analysis showed that participants with SFA intake in the highest quartile had a 23% lower likelihood of PRISm compared to those in the lowest quartile (OR = 0.77; 95% CI: 0.62–0.96). However, the overall trend across quartiles approached but did not reach conventional significance (*P* for trend = 0.055). PUFA intake was also inversely associated with PRISm, with each standard deviation increase corresponding to an odds ratio of 0.88 (95% CI: 0.79–0.99). Analysis across quartiles revealed a significant dose–response relationship, as indicated by a linear trend (*P* for trend = 0.029). In contrast, MUFA did not show a significant association with PRISm in either the continuous model (OR = 1.10; 95% CI: 0.93–1.31) or the quartile-based analysis (*P* for trend = 0.124).

**Figure 4:**
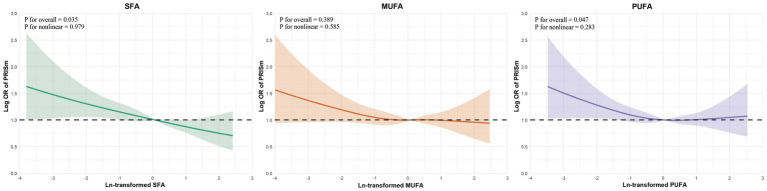
Restricted cubic spline analyses of dietary fatty acids and PRISm. Restricted cubic spline curves for the association between ln-transformed fatty acid intake and odds of PRISm. Shaded areas represent 95% CIs. *p*-values for overall association and nonlinearity are shown in each panel. Models adjusted for Model 3. OR, odds ratio; CI, confidence interval; RCS, restricted cubic spline; PRISm, preserved ratio impaired spirometry.

To further investigate potential nonlinearity in these associations, restricted cubic spline analyses were conducted and visualized in Figure 4. For SFA and PUFA, the associations with PRISm were approximately linear across the full range of intake, as supported by non-significant tests for nonlinearity (SFA: *P* for nonlinearity = 0.979; PUFA: *p* = 0.283). Notably, the overall *p* value for association remained statistically significant for SFA and PUFA. For MUFA, neither the overall nor the nonlinear association with PRISm reached statistical significance (*p* = 0.389 and *P* for nonlinearity = 0.585, respectively), reinforcing the null findings observed in the logistic regression models.

### 3.3 Subgroup analyses

**Figure 5:**
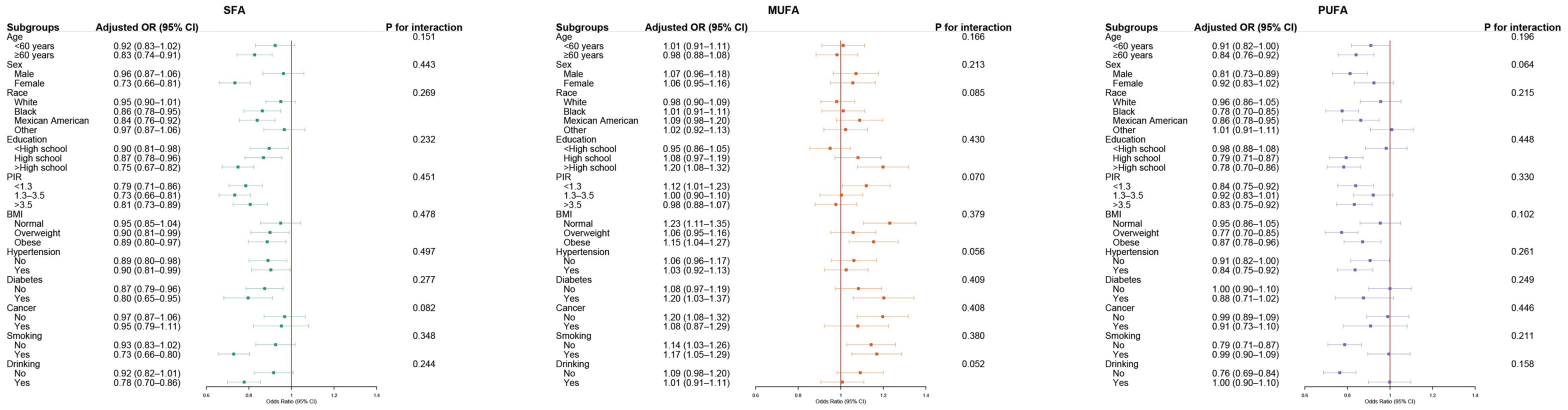
Subgroup analyses of dietary fatty acids and PRISm. Forest plots of adjusted ORs (95% CIs) for PRISm by quartile and per-SD increment of fatty acids across prespecified subgroups (age, sex, race, education, PIR, BMI, hypertension, diabetes, cancer, smoking, drinking). Models adjusted for Model 3. OR, odds ratio; CI, confidence interval; SD, standard deviation; PIR, poverty income ratio; BMI, body mass index.

Subgroup analyses revealed that the inverse associations of both SFA and PUFA intake with PRISm were generally consistent across multiple strata, although the magnitude of association varied (Figure 5). For SFA, stronger inverse associations were observed among participants with diabetes, those who smoked or drank alcohol, and individuals with higher education or PIR. For PUFA, more pronounced associations were noted in older adults, non-Hispanic Black participants, those with obesity, and individuals who drank alcohol. While MUFA intake showed positive associations with PRISm in several subgroups, none of the interaction terms for any fatty acid reached statistical significance (all *P* for interaction >0.05), suggesting no evidence of effect modification.

### 3.4 Sensitivity analyses

To investigate whether the observed associations between fatty acids and PRISm might reflect a broader nutritional deficit, we conducted sensitivity analyses examining total energy and total fat intake. Compared to participants without PRISm, those with PRISm had significantly lower total caloric and fat intake (Supplementary Table S2). In adjusted logistic regression models, the association between total energy intake and PRISm was attenuated and no longer statistically significant (adjusted OR = 0.95 per SD; 95% CI: 0.84–1.08; *p* = 0.420). However, the inverse association between total fat intake and PRISm remained marginally significant (adjusted OR = 0.90 per SD; 95% CI: 0.80–1.01; *p* = 0.070).

We further evaluated the associations between fatty acid intake and lipid profiles to explore potential biological pathways underlying the observed relationships with PRISm. As shown in Supplementary Table S3, higher PUFA intake was significantly associated with higher HDL-cholesterol (+2.6 mg/dL per SD increase; *p* < 0.001) and lower triglyceride levels (−4.3 mg/dL; *p* = 0.010), consistent with an anti-atherogenic profile. Conversely, higher SFA intake was associated with elevated LDL-cholesterol (+4.9 mg/dL; *p* = 0.010) and ApoB (+3.1 mg/dL; *p* = 0.010), while MUFA intake was not significantly related to most lipid markers.

## 4 Discussion

Using nationally representative NHANES data, we found that higher intake of SFA and PUFA was independently associated with lower odds of PRISm, while no significant association was observed for MUFA. These associations remained consistent across adjusted logistic and spline models. Subgroup analyses suggested stronger protective effects in older and non-obese adults, implying potential roles of age-related inflammation or metabolic health in modulating dietary responses.

The stronger associations among older individuals may reflect the influence of age-related low-grade inflammation, or “inflammaging,” which heightens vulnerability to lung function decline and could increase responsiveness to anti-inflammatory dietary components like PUFA (25). Similarly, metabolically healthier, non-obese individuals may exhibit clearer diet–lung association’s due to reduced confounding from systemic inflammation or nutrient imbalance (26, 27).

Previous research has investigated the potential function of dietary fatty acids on pulmonary outcomes, particularly in the context of asthma or COPD (28–34). However, most studies have focused on specific subclasses of fatty acids or on foods that are rich in these components, rather than assessing total fatty acid intake. For instance, Berthon et al. observed that omega-3 PUFA supplementation improved airway inflammation in asthma patients, while Wood et al. demonstrated that higher consumption of fish oil was associated with better lung function in older adults (35, 36). Similarly, Garcia-Larsen et al. identified inverse associations between nut consumption and asthma symptoms in children (37). Despite these findings, such studies often targeted single nutrients or food groups, limiting their generalizability (12). Some studies have attempted a broader assessment of fatty acids and respiratory outcomes (38, 39). In the Hertfordshire Cohort Study, Shaheen et al. reported that individuals with greater PUFA consumption exhibited better lung function and a decreased likelihood of developing COPD (38). Choi et al. reported similar findings in the Korean population, showing beneficial effects of PUFA on lung function and inflammation (39). However, these studies focused on COPD or forced expiratory volume as endpoints, and did not consider PRISm as a distinct phenotype. As far as we are aware, no previous research has comprehensively examined how total intake of saturated, monounsaturated, and polyunsaturated fatty acids relates to the presence of PRISm in individuals from the general population without diagnosed COPD. Unlike previous studies, we used nationally representative NHANES data with standardized dietary assessment and spirometry-based classification of PRISm, enhancing both internal validity and generalizability. As PRISm has been associated with heightened mortality and a greater likelihood of transitioning to overt obstructive pulmonary disease, identifying modifiable dietary contributors may be crucial for early prevention strategies (40, 41).

The observed inverse associations between SFA and PUFA intake and PRISm may be biologically plausible through several pathways. Both saturated and polyunsaturated fats play key roles in modulating systemic inflammation, oxidative stress, and lipid homeostasis—mechanistic pathways closely connected to respiratory health. Polyunsaturated fatty acids, especially the omega-3 subclass abundant in sources such as oily fish, walnuts, and flaxseeds, have been reported to attenuate inflammatory responses by modulating the synthesis of eicosanoids, including prostaglandins and leukotrienes, as well as influencing cytokine signaling (42, 43). These mechanisms may attenuate subclinical inflammation in the lungs, thereby preserving pulmonary function (44). Additionally, experimental and clinical studies have provided further mechanistic support. For example, omega-3 PUFAs have been shown to downregulate the production of pro-inflammatory eicosanoids such as leukotriene B4 and prostaglandin E2, and to suppress cytokines including TNF-*α* and IL-6 through activation of peroxisome proliferator-activated receptor gamma (PPARγ) (45–47). Clinical evidence has also demonstrated that omega-3 supplementation improves airway inflammation and lung function in asthma patients (45). Furthermore, dietary fatty acids influence surfactant composition, a critical determinant of alveolar stability and gas exchange. Surfactant phospholipids, enriched in polyunsaturated fatty acids, maintain membrane fluidity and suppress excessive immune responses in alveolar macrophages (48, 49). Disruption in surfactant lipid balance, particularly an increase in cholesterol or saturated lipid fractions, has been linked to impaired lung compliance and inflammatory activation. Lastly, omega-3 PUFAs may reduce oxidative burden through upregulation of antioxidant pathways, including glutathione peroxidase and PPARγ-mediated transcription, potentially offering protection against oxidative injury to pulmonary tissues (50, 51). Conversely, while SFAs have often been viewed as pro-inflammatory, emerging evidence suggests that not all SFAs exert detrimental effects and their impact may vary depending on food source and overall dietary context (52, 53). In our study, the primary sources of SFA were dairy and unprocessed meats, which may have different metabolic consequences compared to industrial trans fats or processed food-derived SFAs. Notably, the observed inverse association between saturated fat intake and PRISm contrasts with the traditionally held view that SFAs are harmful for respiratory or metabolic health. However, there is increasing recognition of heterogeneity in the health effects of SFAs depending on their origin. For instance, SFAs from dairy products may be accompanied by beneficial nutrients such as calcium, vitamin D, and specific bioactive lipids that modulate inflammation and metabolism. Indeed, several population-based studies have reported neutral or even protective associations between dairy-based SFA intake and cardiometabolic outcomes (48, 54).

Moreover, saturated fat intake may reflect broader dietary adequacy in our population. Participants with PRISm exhibited lower total energy and nutrient intake, which could indicate underlying malnutrition or frailty—both of which are associated with impaired pulmonary function. A study by Cornell et al. in patients with COPD found that individuals with the lowest quartile of SFA intake had significantly worse lung function compared to those with moderate or high intake, suggesting that insufficient rather than excessive SFA intake may be detrimental in certain populations (49). Although our models adjusted for total energy intake and multiple covariates, residual confounding by unmeasured dietary factors—such as micronutrient intake, protein quality, or overall diet pattern—cannot be excluded. Therefore, the observed inverse association between SFA intake and PRISm should be interpreted with caution and may reflect complex interactions between dietary quality, metabolic health, and pulmonary function.

Beyond local inflammatory or oxidative pathways, our additional analyses suggest that the protective association of PUFA intake with PRISm may be partly mediated through favorable systemic lipid metabolism. Specifically, we found that higher PUFA intake was significantly associated with increased HDL-cholesterol and reduced triglyceride levels—two biomarkers that have been positively associated with lung function measures such as FEV₁ and FVC in previous epidemiologic studies (55, 56). Conversely, higher SFA intake was linked to elevated LDL-cholesterol and apolipoprotein B, which are implicated in impaired pulmonary function and increased emphysema burden (57). These observations align with mechanistic studies showing that cholesterol transport and lipoprotein imbalance may influence pulmonary inflammation, surfactant composition, and alveolar macrophage function (58). Taken together, these findings suggest that dietary fat composition may influence lung function not only through direct anti-inflammatory or antioxidant effects, but also by modulating lipid homeostasis and systemic metabolic health. Public health strategies emphasizing the consumption of polyunsaturated over saturated fats may therefore contribute to improved pulmonary outcomes through multiple complementary pathways. Previous studies have also implicated lipid metabolism and body composition in respiratory outcomes, particularly in the context of asthma and COPD. For example, Wang et al. conducted a cross-sectional and bidirectional Mendelian randomization study using NHANES data and found that greater fat mass—measured across various body compartments—was significantly associated with increased asthma risk in adults (59). These findings underscore the importance of metabolic dysregulation and adipose-related inflammation in pulmonary pathophysiology. Similarly, Ischaki et al. showed that fat-free mass index (FFMI), rather than BMI alone, was more strongly correlated with airflow limitation, dyspnea, and functional capacity among COPD patients, highlighting the relevance of body composition and nutritional status to disease progression (60). In our study, we found that higher PUFA intake was linked to favorable lipid profiles (e.g., higher HDL-C, lower triglycerides), while SFA intake was associated with increased LDL-C and ApoB concentrations. These markers have previously been associated with lung function measures such as FEV₁ and FVC, and may partly mediate the observed associations between dietary fat composition and PRISm. Together, these observations reinforce the potential role of lipid homeostasis and body composition in maintaining respiratory health.

Furthermore, lipid metabolism and surfactant homeostasis are tightly linked to pulmonary structure and function (61, 62). Adequate fatty acid intake may support alveolar integrity and membrane composition, which are essential for optimal gas exchange and lung compliance (63). Oxidative stress, another key contributor to lung dysfunction, can also be modulated by dietary fatty acid balance. PUFA, in particular, may reduce oxidative burden via upregulation of antioxidant pathways such as glutathione peroxidase or PPARs (64).

From a public health perspective, our results support dietary strategies that emphasize nutrient-dense sources of PUFA and SFA, such as fish, nuts, and dairy, which may help preserve lung function even before the onset of symptomatic disease. As PRISm often goes undiagnosed, identifying modifiable risk factors is crucial for early intervention. These findings may inform dietary guidelines for respiratory health and support cost-effective prevention strategies targeting at-risk groups.

Future studies should explore causality using prospective cohorts and interventional designs. Investigating dietary interventions (e.g., omega-3–rich foods) or integrating biomarker and microbiome data may uncover relevant mechanisms. Expanding research to diverse or younger populations is also essential to assess generalizability and inform tailored recommendations.

This study benefits from the use of a large, nationally representative dataset, incorporating high-quality spirometry, detailed dietary assessments, and comprehensive clinical information collected through standardized protocols. The use of both continuous and categorical models, as well as non-linear spline regression, provides a comprehensive evaluation of dose–response relationships. Furthermore, the inclusion of extensive adjustments for sociodemographic, behavioral, and clinical variables, along with the application of a validated machine learning–based imputation technique for missing data, enhances the robustness of our findings and reduces the potential for bias due to incomplete information.

Several limitations merit consideration. First, as this was a cross-sectional analysis, it does not allow for conclusions regarding causal links between dietary fatty acid consumption and PRISm (65). Second, dietary data were obtained through self-reported 24-h recalls, which, although collected using a validated multiple-pass method, remain susceptible to recall inaccuracies and reporting bias. Third, residual confounding from unmeasured factors such as physical activity, supplement use, or occupational exposures cannot be fully ruled out. Finally, although we applied a validated machine learning–based imputation method to handle missing data, while such methods help to preserve statistical power and reduce bias compared to complete-case analysis, they may still introduce uncertainty if the missingness mechanism is not fully captured.

## 5 Conclusion

In conclusion, this study suggests that higher consumption of saturated and polyunsaturated fatty acids is associated with a reduced prevalence of PRISm among U.S. adults. These results highlight the potential importance of dietary fatty acid composition in maintaining lung health, particularly among individuals without overt airflow obstruction. If confirmed by future prospective studies and clinical trials, these findings may support the development of nutritional strategies that emphasize increased intake of polyunsaturated fats, for example from fish, nuts, and seeds, and potentially moderate intake of saturated fats from specific sources such as dairy products. However, given the cross-sectional nature of the study, causal interpretations should be made with caution. Further longitudinal and mechanistic investigations are warranted to elucidate these associations and to inform evidence-based dietary recommendations for respiratory health.

## Data Availability

The raw data supporting the conclusions of this article will be made available by the authors, without undue reservation.
