# Supplementary material for: Association between dietary fatty acid intake and preserved ratio impaired spirometry in U.S. adults: a population-based cross-sectional study
Source: Front Nutr. 2025 Jul 30;12:1622795. doi: 10.3389/fnut.2025.1622795 (PMC12343235; doi:10.3389/fnut.2025.1622795)
Supplement: Supplementary file 2 [file Table_2.docx]

**Supplementary Table S2**. Associations between total energy and total fat intake with PRISm.

| **Exposure Variable** | **Model** | **OR for PRISm**  **(per 1-SD increase)** | **95% CI** | **P-value** |
| --- | --- | --- | --- | --- |
| Total energy intake | Unadjusted | 0.81 | 0.72–0.90 | <0.001 |
|  | Adjusted¹ | 0.95 | 0.84–1.08 | 0.420 |
| Total fat intake | Unadjusted | 0.85 | 0.76–0.94 | 0.002 |
|  | Adjusted¹ | 0.90 | 0.80–1.01 | 0.070 |

¹ Adjusted for age, sex, race/ethnicity, education level, PIR, BMI, smoking status, alcohol use, comorbidities (hypertension, diabetes, cancer).
