# Supplementary material for: Association between dietary fatty acid intake and preserved ratio impaired spirometry in U.S. adults: a population-based cross-sectional study
Source: Front Nutr. 2025 Jul 30;12:1622795. doi: 10.3389/fnut.2025.1622795 (PMC12343235; doi:10.3389/fnut.2025.1622795)
Supplement: Supplementary file 3 [file Table_3.docx]

**Supplementary Table S3**. Associations between fatty acid intake and serum lipid biomarkers

| **Exposure (per 1-SD increase)** | **HDL-C (mg/dL)** | **LDL-C (mg/dL)** | **Triglycerides (mg/dL)** | **Total Cholesterol (mg/dL)** | **ApoB (mg/dL)** |
| --- | --- | --- | --- | --- | --- |
| Saturated fat (SFA) | −0.2 (p = 0.51) | +4.9 (p = 0.01) | +1.7 (p = 0.09) | +5.3 (p = 0.02) | +3.1 (p = 0.01) |
| Monounsaturated fat (MUFA) | +0.5 (p = 0.32) | +1.7 (p = 0.14) | −1.2 (p = 0.23) | +1.6 (p = 0.20) | +1.2 (p = 0.17) |
| Polyunsaturated fat (PUFA) | +2.6 (p < 0.001) | −1.1 (p = 0.36) | −4.3 (p = 0.01) | +0.3 (p = 0.85) | −1.9 (p = 0.11) |

All models adjusted for age, sex, race/ethnicity, PIR, BMI, smoking status, alcohol intake, and total energy intake.
